# Unexploited Antineoplastic Effects of Commercially Available Anti-Diabetic Drugs

**DOI:** 10.3390/ph9020024

**Published:** 2016-05-06

**Authors:** Panagiota Papanagnou, Theodora Stivarou, Maria Tsironi

**Affiliations:** Department of Nursing, Faculty of Human Movement and Quality of Life Sciences, University of Peloponnese, Orthias Artemidos and Plateon St, Sparti GR-23100, Greece; theodorast@gmail.com (T.S.); mtsironi@otenet.gr (M.T.)

**Keywords:** drug repurposing, tumorigenesis, anti-diabetic agents, applicability

## Abstract

The development of efficacious antitumor compounds with minimal toxicity is a hot research topic. Numerous cancer cell targeted agents are evaluated daily in laboratories for their antitumorigenicity at the pre-clinical level, but the process of their introduction into the market is costly and time-consuming. More importantly, even if these new antitumor agents manage to gain approval, clinicians have no former experience with them. Accruing evidence supports the idea that several medications already used to treat pathologies other than cancer display pleiotropic effects, exhibiting multi-level anti-cancer activity and chemosensitizing properties. This review aims to present the anticancer properties of marketed drugs (*i.e.*, metformin and pioglitazone) used for the management of diabetes mellitus (DM) type II. Mode of action, pre-clinical *in vitro* and *in vivo* or clinical data as well as clinical applicability are discussed here. Given the precious multi-year clinical experience with these non-antineoplastic drugs their repurposing in oncology is a challenging alternative that would aid towards the development of therapeutic schemes with less toxicity than those of conventional chemotherapeutic agents. More importantly, harnessing the antitumor function of these agents would save precious time from bench to bedside to aid the fight in the arena of cancer.

## 1. Introduction

Pleiotropy in biological systems is a long known phenomenon, commonly attributed to the complexity of intracellular signaling networks or tissue-specific cellular responses [1,2,3,4]. Consequently, a growing list of pharmacological agents is being reported to exhibit therapeutic potential in pathologic entities that are not mechanistically relevant with their current therapeutic use [5,6,7]. Besides, the involvement of non-antitumor compounds with cancer-associated networks corroborates the notion that their use could be expanded in the field of oncology aside from their current use [8,9,10,11]. This is widely known as drug “repurposing” or “repositioning” in oncology. Paying credence to this emerging concept, diverse commercially available non-antineoplastic agents such as anti-hypercholesterolemic agents [12], nonsteroidal anti-inflammatory drugs (NSAIDs), agents currently used for the management of hypertension or even agents received by patients suffering from acute immunodeficiency syndrome (AIDS) exhibit profound anti-cancer activity [13,14,15].

Herein, the antitumor activity of the marketed anti-diabetic drugs metformin and pioglitazone is reviewed. This is evidenced either by pre-clinical *in vitro* and *in vivo* data or clinical data from studies in DM type II patients or nondiabetic individuals. Despite the fact that many anti-diabetic medications are currently available in the market, only the biguanide metformin and the thiazolidinedione (TZD) pioglitazone are mentioned. This is why insulin administered for the management of DM type I and insulin secretagogues (sulfonylureas) have been associated with an increased incidence of cancer [16,17,18]. Studies regarding the correlation (either positive or negative) among glucagon-like peptide 1 (GLP-1)-based medications including dipeptidyl peptidase 4 (DDP-4) inhibitors (the so-called “gliptins”) or anti-diabetics that target renal sodium-glucose cotransporter 2 (SGLT2 inhibitors or “gliflozins”) and cancer, cannot be considered as conclusive [19,20]. On the other hand, only little evidence has been provided for the anti-tumor properties of non-sulfonylurea secretagogues (known as “glinides”) or α-glucosidase inhibitors [21,22] whereas the TZDs rosiglitazone and troglitazone that fall into the same category of drugs which exhibit profound anti-tumor activity [23,24,25,26,27] have been withdrawn from the market [28] due to their cardiotoxicity and hepatotoxicity, respectively. This is also the case for the biguanide phenformin that also exhibits anti-cancer properties [29,30,31], but is no longer commercially available because of its severe adverse lactic acidosis effect [32].

## 2. Metformin and Pioglitazone: Overview of Current Clinical Use and Molecular Targets

Metformin is a first-line anti-diabetic agent [33] widely prescribed all over the world. It acts as an insulin sensitizer and it can be used either as monotherapy or as part of combinational formulations. Metformin can also prevent the development of diabetes in subjects diagnosed with prediabetes [34]. However, the formal use of metformin is only for the treatment of diabetes. Pioglitazone is also used for the treatment of DM type II [35] and can be administered alone or in conjunction with other anti-diabetics including metformin or sulfonylurea analogues.

There is convincing evidence for a direct correlation between DM type II (also called adult-onset or non-insulin-dependent DM) and cancer [36,37,38,39], particularly postmenopausal breast cancer [40,41]. Notably, patients with DM type II run a 10%–20% greater risk than non-diabetic females for developing breast cancer while up to 16% of breast cancer patients are diabetics [42]. In addition, DM type II is associated with worse prognosis and poor outcome of breast cancer [43]. DM type II is a metabolic disorder characterized by the disturbed blood glucose control, insulin resistance and hyperinsulinemia [36]. The latter clinical finding, in turn, is linked with the pathogenesis of cancer due to the mitogenic activity of insulin [36,37,44]. Yet, recent evidence indicates that the anti-cancer properties of metformin are largely attributed to cell autonomous mechanisms [32].

**Figure 1:**
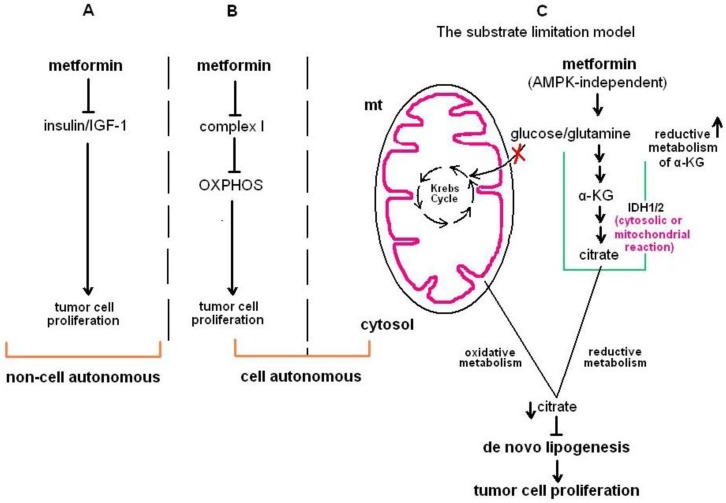
Models that have been proposed to explain the inhibition of tumor cell proliferation by metformin. (**A**) metformin prevents tumor cell proliferation in a cell non-autonomous fashion through blocking the insulin/insulin-like growth factor 1 (IGF-1) signaling axis. (**B**) Metformin-induced blockage of tumor cell proliferation is cell autonomous and is mechanistically associated with the inhibition of complex I of the oxidative phosphorylation (OXPHOS). (**C**) The substrate limitation model. According to this model, metformin acts in an AMPK-independent manner to block the usage of glucose and glutamine by oxidative reactions (Krebs cycle) in mitochondria (mt) of tumor cells. Besides, it promotes a shift towards reductive rather than oxidative α-ketoglutaric acid (α-KG) metabolism. The reductive carboxylation of glutamine-derived α-KG that takes place either in cytosol (mediated by isocitrate dehydrogenase 1, IDH1) or in mitochondria (mediated by IDH2) is being boosted by metformin. Although the presence of metformin favors the production of citrate the reductive carboxylation of α-ΚG, the hindrance of OXPHOS in mitochondria induced by the drug results in a decrease of total citrate derived from either mitochondrial or cytosolic reactions. The drop of lipogenic citrate leads in the prevention of tumor cell proliferation which requires de novo lipogenesis. The red “X” symbol denotes inhibition of a signaling pathway while upward and downward pointing arrows denote up- and downregulation, respectively.

Metformin acts as an activator of AMP-activated protein kinase (AMPK) which serves as a master metabolic sensor and is a negative modulator of the mammalian target of rapamycin (mTOR) [45]; a point of convergence for tumorigenesis and energy homeostasis [46]. AMPK and its upstream activator, the LKB1 tumor suppressor, are thought to play a central role in the anti-cancer function of metformin [47,48]. However, metformin can also stimulate AMPK-independent pathways which halt cancer cell proliferation [49,50] or it may engage an AMPK-dependent/LKB1-independent pathway to suppress the proliferation of malignant cells [51]. To date, it has been suggested that the antiproliferative activity of metformin can be attributed either to its ability to impair insulin/IGF-1-mediated signaling or its inhibitory activity of complex I of oxidative phosphorylation [52,53]. However, recent data support a “substrate limitation” model (Figure 1) in order to explain the ability of metformin to suppress tumor cell proliferation. According to this model, metformin owes its antitumor activity to the inhibition of lipogenic citrate production via the oxidative metabolism pathway (lipogenic processes are crucial for the synthesis of membranes and tumor cell proliferation) in mitochondria due to drug-induced depletion of Krebs cycle intermediates in an LKB1- and AMPK-independent manner. Lack of functional mitochondria endows tumor cells with insensitivity to metformin. In these cells, sensitivity to the cytostatic effects of metformin can be restored via silencing ATP citrate lyase (ACL; the enzyme catalyzing the rare limiting step of acetyl-CoA production), pinpointing to the significance of future therapeutic strategies employing metformin along with ACL activity inhibition. On the other hand, metformin increases the production of citrate via a reductive route (the reductive carboxylation of α-ketoglutaric acid). However, this type of metabolic shift is not sufficient to sustain tumor cell proliferation in the presence of metformin [54]. 

On the other hand, pioglitazone serves as an activator of the peroxisome proliferator-activated receptor γ (PPARγ). This nuclear receptor is widely expressed in human epithelial tissues and apart its role in glucose/lipid homeostasis it is also critically involved in cell differentiation and inhibition of cell growth [26,55]. Both metformin and pioglitazone have been found to modulate metabolic routes that are critical for tumor cell biology [56,57,58]. This is consistent with the well-known connection between altered energy metabolism and cancer [59,60,61].

### 2.1. Pre-Clinical Data Suggesting Possible Repurposing

#### 2.1.1. *In Vitro* Evidence for the Antineoplastic Effects of Metformin

*In vitro* experimentation suggests a plausible repositioning of metformin in the field of gynecologic oncology. Metformin is known to decrease the proliferative capacity and clonogenicity of breast cancer cell lines, regardless of p53 status and the status of estrogen and ErbB2 (elsewhere also referred to as Her-2/neu) receptors [62]. In the latter study, metformin negatively influenced the expression or the activity of various cell cycle- and cell growth-regulatory molecules exemplified by E2F1, MAPK, AKT and mTOR. Low-dose metformin was reported to display a selective cytotoxicity over cancer stem cells (CSCs) in breast cancer types with different genetic background [63]. This finding pays further credit to the validity of CSC theory. The suppressive effects of metformin on the biosynthetic pathway of estrogens both in breast cancer cells [64] and in adipocytes in breast tissue [65] underline its multi-level anti-cancer function encompassing the targeting of pathways operating not only in malignant elements themselves but also in stromal cells in the tumor microenvironment [29,66]. Unfortunately, recent evidence suggests that the prolonged exposure of estrogen receptor (ER)-positive human breast cancer cells to metformin upregulates AKT/Snail1, suppresses ER and renders these cells tolerant to the toxicity of both metformin and tamoxifen; a phenomenon known as “cross-resistance”, irrespective of AMPK stimulation [67]. In ovarian cancer cells metformin has been found to exert cytostatic effects [68]. Consistent with data demonstrating the ability of metformin to eliminate ovarian CSCs [69], it was reported that low-dose metformin restrains the self-renewing capacity of CD44/CD117-positive ovarian CSCs as well as the expression of epithelial-to-mesenchymal transition (EMT) markers *in vitro* [70]. Besides, irrespective of ER status, metformin exerts anti-EMT effects on 17β-estradiol-treated human endometrial adenocarcinoma cells via engaging a βKlotho/ERK-dependent pathway. These effects partially depend on AMPKα [71].

Metformin displays antitumor properties not only in studies using breast cancer and ovarian cancer cells, but also in a series of experiments with other types of malignant cells. In human pancreatic cancer cells metformin acts in a cytostatic manner. Drug-induced cytostasis in these cells coincides with an alteration in the expression profile of microRNAs and cell cycle-modulatory molecules [72]. On the other hand, non-small cell lung cancer (NSCLC) cells relay on Nemo-like kinase (NLK) for their stemness and their ability to proliferate and metformin has been found to inhibit this kinase, thereby suppressing both NSCLC cell proliferation and stemness [73]. LKB1 seems to be dispensable for the anti-proliferative activity of metformin in NSCLC cells [51]. The finding that in NSCLC H1299 cells metformin counteracts the biosynthetic processes that depend on mitochondrial reactions [50] propelled the suggestion of the “substrate limitation” model (Figure 1), as mentioned above.

Head and neck cancer (HNC) encompasses different pathological entities including nasopharyngeal carcinoma (NPC) and head and neck squamous cell carcinoma (HNSCC), with HNSCC patients running the risk to develop second primary esophageal squamous cell carcinoma (ESCC) [74]. Metformin has actually been demonstrated to exhibit antitumor activity both in HNC cells and in studies assessing its antineoplastic properties in esophageal cancer *in vitro*. Significantly, the expression of the organic cation transporter 3 (OCT3) which mediates the uptake of metformin into HNSCC cells is necessary for the drug-induced growth suppression via the inhibition of the mTOR pathway [75]. In esophageal adenocarcinoma (EAC) or nasopharyngeal carcinoma cells the antitumorigenicity of metformin is associated with the modulation of the expression of cell cycle-regulatory proteins [76] and DNA repair factors [77], respectively. On ESCC cells, metformin exerts cytostatic effects in an AMPK-dependent fashion [78].

Metformin, apart from its growth-inhibitory properties has also been reported to affect cancer cell viability. Actually, metformin triggers apoptosis *in vitro*, *i.e.*, in gastric [79], pancreatic [80], colon cancer [47] and salivary adenocarcinoma cells [81]. P53-null colon cancer cells are presumed to be susceptible to metformin-induced apoptosis owing to their inability to undergo the metabolic alterations imposed by metformin in the absence of p53 which is a crucial controller of various aspects of metabolism [47]. This finding is of great importance because it highlights the therapeutic potential of metformin in the treatment of p53-deficient tumors. Further, metformin in combination with the glycolytic inhibitor 2-deoxyglucose (2-DG) makes the prostate cancer cell’s life/death decision scale to tilt towards apoptosis whereas metformin or 2-DG alone exert minor cytotoxicity [82]. Similarly, 2-DG potentiates the toxic effects of metformin in thyroid cancer cells [83]. In prostate cancer cells metformin also negatively modulates lipogenesis; an anabolic route which is a key characteristic of tumor cells [84]. These studies pave the road for the maximal exploitation of metabolic modulators including metformin in cancer therapeutics. Metformin promotes the apoptosis of quiescent B-cell chronic lymphocytic leukemia (CLL) cells. Also, this drug prevents CLL cells from entering the cell cycle upon their stimulation through co-culture with CD40L-positive fibroblasts [85]. In highly invasive C4-2B cells metformin in conjunction with simvastatin evokes necrosis, thereby circumventing the resistance to apoptosis characterizing these prostate cancer cells [58]. In hepatocellular carcinoma (HCC) cells metformin functions as a radiosensitizer via increasing oxidative stress [86] while in osteosarcoma cells metformin potentiates the cytotoxic effects of cisplatin [87].

Besides, metformin influences the migratory/metastatic activity of various cancer cells. Metformin can possibly inhibit the metastatic potential of prostate cancer cells through upregulating miR30a that, in turn, prevents an EMT program mediated by its target SOX4 [88]. The invasive and migratory potential of prostate cancer cells is also decreased by metformin in prostate cancer cell via the impairment of the insulin-like growth factor 1 receptor (IGF-1R) axis [89]. The metformin-induced suppression of the EMT has been described in thyroid cancer cells, too. In this case, this metformin’s function has been attributed to its ability to inhibit the kinase mTOR [90]. In addition, metformin dampens the proliferative as well as the invasive potential of MG63 osteosarcoma cells and counteracts their stemness [91]. The invasiveness of B16F10 mouse melanoma cells is decreased by metformin due to up-regulation of E-cadherin expression [92].

#### 2.1.2. Pre-Clinical *In Vivo* Evidence for the Antineoplastic Effects of Metformin

There is a considerable amount of *in vivo* evidence of the preclinical anti-neoplastic activities of metformin, as presented immediately below. Data from an animal model of mammary tumor virus (MMTV)-ErbB2 tumorigenesis underscore the preventive antitumor function of metformin, since it selectively inhibits the proliferation of a specific cellular subpopulation which is being incriminated for tumor initiation; those with the CD61^(high)/^CD49f^(high)^ immunophenotype. Importantly, the “Achilles heel” of the tumor-initiating cells (TIC)/CSC-rich tumorspheres was shown to be the elevated ErbB2 whose expression is ablated by metformin [93]. A similar CSC-killing activity was reported for metformin in ErbB2-positive mouse xenografts where metformin-mediated toxicity towards human CD44^+/^CD24^−/low^ breast CSCs is associated with sensitization to the humanized anti-ErbB2 monoclonal antibody trastuzumab [94]. Of note, metformin displays antitumor synergy with several conventional chemotherapeutic agents aside from trastuzumab. In addition, it does not only prevent tumor initiation as mentioned above, but also it prevents the relapse of cancer [33]. Moreover, metformin prolongs the survival of murine ErbB2 transgenes [95] and exhibits chemosensitizing properties in breast cancer cell line xenografts [63]. Also, metformin in synergy with everolimus restrains the growth of tumors from xenografts of HCC1428 breast cancer cells [96].

Pre-clinical, *in vivo* evidence for the antitumor properties of metformin has been also provided for glioblastoma [97], esophageal cancer [76,78,98] as well as prostate cancer [89], ovarian cancer [68] and salivary gland adenocarcinoma [81] in mouse models. In fact, metformin inhibits the growth of ESCC xenograft mouse models; an event which is molecularly associated with the upregulation of Cip/Kip family members that are known to perturb cell cycle progression [78]. Significantly, metformin inhibits the growth of human pancreatic cancer xenografts, possibly due to the ablation of the crosstalk among an insulin receptor (IR) and G protein-coupled receptors (GPCRs), in an AMPK-dependent manner [99]. In addition, in human pancreatic cancer xenografts, metformin causes tumor cells to cease proliferation and impinges on the expression of microRNAs as well as cell cycle-regulatory molecules [72]. *In vivo* evidence for the AMPK-dependent antitumoral activity of metformin associated with mTOR inhibition was also provided in murine models of chemical-induced colorectal carcinogenesis where metformin was found to suppress the formation of ACFs [100]. In nude mice with lung adenocarcinoma metformin inhibits the occurrence of distant metastases, possibly through opposing the phenomenon of EMT orchestrated by an IL-6/STAT3 axis [101]. Of note, in Ewing sarcoma xenografts adequate tumor oxygenation is essential for the unperturbed metformin-dependent activation of AMPK which possibly mediates the anti-growth function of metformin [102]. In PC-3 xenograft models of human prostate carcinoma, tumor growth is suppressed via the impaired transcription of the gene coding for IGF-1R [89].

A marked antitumor synergy among metformin and paclitaxel has been found in transgenic animal models of ovarian cancer since tumors exposed to both of these agents are 60% lighter than those solely exposed to paclitaxel or metformin [68]. Metformin exhibits anti-growth effects on SKOV3 xenografts where in synergy with cisplatin diminishes the subpopulation of ovarian CSCs with the CD44+/CD117+ immonophenotype. Further, metformin displays anti-EMT properties in this subpopulation at a dose of 0.1 mM [70]. This is of major importance given the association of EMT with stemness and their impact on cancer therapeutics and prognosis [103]. In HCC, metformin can be combined with the multikinase inhibitor sorafenib in order to mitigate sorafenib-induced down-regulation of the tumor suppressive protein TIP30. In fact, metformin and sorafenib in combination interfere with a partially AMPK-dependent TIP30/thioredoxin pathway to prevent extrahepatic metastases [104].

#### 2.1.3. *In Vitro* Evidence for the Antineoplastic Effects of Pioglitazone

A series of *in vitro* studies are suggestive of the future exploitation of pioglitazone for the therapy of upper gastrointestinal (GI) tract cancers (pancreas and liver cancer), gynecologic cancer (breast cancer), primary brain tumor (glioma) as well as hematological tumors. A drug-induced downregulation of the transcription of cyclooxygenase-2 (COX-2) and interleukin-8 (IL-8) was reported throughout the course of *in vitro* experiments using pioglitazone and the proliferation of various pancreatic cancer cells was halted [105]. In bioptic material from HCC patients the expression of receptor for advanced end glycation products (RAGE) is elevated compared with the expression of this receptor in tumor adjacent healthy tissue, as evidenced by tissue microarrays (TMAs). In HCC cells, short interference RNA (siRNA)-mediated RAGE silencing phenocopies the anti-proliferative and anti-invasive effects of pioglitazone. These findings suggest a role for RAGE in the pathophysiology of HCC and a RAGE-dependent mechanism for the antitumor activity of pioglitazone [106]. In human preadipocytes (obesity in postmenopausal women has been linked to hormone-dependent breast cancer) pioglitazone negatively controls the levels of aromatase via derepressing its 15-hydroxyprostaglandin dehydrogenase (15-PGDG)-mediated degradation as well as by inducing its positive regulator, the BRCA1 tumor-suppressor. This finding favors the notion that pioglitazone might substitute aromatase inhibitors in breast cancer prevention or treatment. This is of major clinical importance since aromatase inhibitors have been linked to iatrogenic osteoporosis [107]. The anti-invasive effects of pioglitazone on the highly metastatic breast cancer cell line MDA-MB-231 are mitigated by the indole MK886 although this compound exhibits MDA-MB-231 cell-killing properties [108]. This is consistent with previous data demonstrating that MK886 prevents PPAR-dependent signaling [109]. In glioma cells pioglitazone engages a β-catenin-dependent route to impede their growth and decrease their invasiveness [110]. Moreover, it has been demonstrated that pioglitazone exhibits selective anti-growth activity against leukemic rather than normal human hematopoietic progenitor cells [111]. The latter finding opens the possibility of a future exploitation of pioglitazone in the field of leukemia therapeutics.

#### 2.1.4. Pre-Clinical *In Vivo* Evidence for the Antineoplastic Effects of Pioglitazone

Pioglitazone was found to suppress the growth of BxCP-3 xenografts in mice and to inhibit their ability to give rise to lymph node and distal (pulmonary) metastases [105]. In a mouse model of prostate cancer, pioglitazone significantly reduces the occurrence of bone metastasis at the clinically achievable dose of 30 mg/kg/day in synergy with the histone deacetylase (HDAC) inhibitor valproic acid. This anti-metastatic activity of pioglitazone is possibly attributed to its ability to up-regulate the expression of E-cadherin through a peroxisome proliferator response element (PPRE) within its promoter region which becomes PPARγ-responsive only in the presence of HDAC inhibitors. The therapeutic applicability of such a combinational scheme is substantiated by the findings that *E*-cadherin down-regulation is a common event in prostate cancer whereas the expression of PPARγ is elevated in this type of malignancy [112]. In animal models, pioglitazone was also found to inhibit the early-stage chemical-induced hepatocellular carcinogenesis [113] and to suppress tumor growth via antiangiogenic mechanisms in human NSCLC xenografts in mice [114]. Further, there has been provided *in vivo* evidence for a pyruvate dehydrogenase kinase 4 (PDK4)-dependent pathway activated by pioglitazone by which this PPARγ ligand increases the oxidative stress experienced by lung cancer cells, thereby ceasing their proliferation [57].

In the KK-Ay mouse model for DM type II and obesity pioglitazone both improves several metabolic parameters and impairs the development of precancerous lesions in colon (aberrant crypt foci, ACFs) upon exposure to the potent carcinogen azoxymethane. This finding suggests the chemopreventive value in colorectal tumorigenesis [115]. Importantly, a former study had shown that pioglitazone inhibits liver metastasis in human colon cancer xenografts [116], indicating an additional anti-metastatic role of pioglitazone.

### 2.2. Clinical Data Suggesting Possible Repurposing

#### 2.2.1. Metformin

A large body of literature indicates that the usage of metformin both lowers the incidence of various types of cancer (e.g., hepatocellular carcinoma, pancreatic cancer, lung cancer, gynecological cancer and prostate cancer) in diabetics [17,18,21,117,118,119,120,121,122,123,124,125,126] and decreases the mortality in cancer sufferers with DM type II [127,128,129,130]. In accordance with data from experimentation in animals [100], a pilot study argued for the preventive role of metformin in human colorectal carcinogenesis: short-term, low-dose (250 mg/d) metformin treatment in nondiabetic subjects with rectal ACFs caused a marked decrease in ACFs per patient [131]. In addition, a population-based case-control study showed that peri- and postmenopausal Danish women with DM type II receiving metformin run a lower danger to develop breast cancer than non-users [132]. In contrast, this is not the case for other anti-diabetic drugs such as insulin secretagogues and insulin itself [133,134,135]. Rather, sulfonylureas and insulin have been associated with increased risk for cancer [17,18].

Retrospective studies reviewing the clinical outcome of HER2-positive breast cancer patients with diabetes suggest that metformin beneficially affects their cancer-specific survival [136,137]. Another study, however, reported that the overall survival is not influenced by metformin in triple-negative breast cancer (TNBC) diabetics receiving adjuvant chemotherapy. Still, metformin manages to lower the risk for the occurrence of distal metastases [138]. A population-based study which recruited breast cancer women with or without DM treated with neoadjuvant chemotherapy demonstrated a significant increase in the rate of pathologic complete response (pCR) among the diabetics who received metformin and those who did not [139]. However, retrospective analysis of datasets regarding the cancer-specific outcome in chemotherapy-receiving women suffering from DM type II and invasive breast cancer indicates that the intake of metformin is not associated with any improval [140]. Data favouring the notion of the antitumor activity of this antidiabetic agent are also available for diabetic patients with esophageal cancer [98], prostate cancer [133] as well as in women with endometrial cancer where it preoperatively halts DNA replication in serum, at clinically attainable dosage [141]. A small phase I clinical study in advanced solid tumor patients who were administered metformin in conjunction with the mTOR inhibitor temsirolimus reported disease stabilization in 5 out of 11 participants. Unfortunately, when combined even at clinically relevant doses (500 mg of metformin twice a day and 25 mg/week of temsirolimus) these two medications caused toxic effects; something which limits the potent future therapeutic value of such a scheme [142]. Unfortunately, a recent randomized phase II clinical study did not reported any statistically significant difference among the survival rates of patients in The Netherlands suffering from advanced pancreatic cancer who received metformin or placebo combined with erlotinib *per os* and intravenous gemcitabine, at six months [143].

Altogether, metformin seems to be a promising chemopreventive agent in breast and colorectal cancer. However, although there is ample evidence for the antitumor function of metformin principally stemming from population-based case-control studies and retrospective studies as extensively has been elsewhere reviewed [144], more evidence is needed from phase II studies whereas no data from a large phase II and III trial have been published so far. In addition, many clinical studies have yielded inconclusive results [145]. The completion of ongoing clinical trials (e.g., NCT01243385 and NCT01627067) assessing the anti-cancer utility of metformin is therefore much-awaited.

#### 2.2.2. Pioglitazone

Objective responses and stabilization of disease were observed in a phase II clinical trial recruiting patients with chemorefractory melanoma and soft tissue sarcoma who were treated with a combinational scheme of metronomic therapy using trofosfamide, rofecoxib (a COX-2 inhibitor which is no more commercially available) and clinically relevant doses (45 mg/day) of pioglitazone [146]. Encouraging results (disease stabilization or even complete remission) have been noted in a pilot study where the participants suffered from advanced vascular malignancies [147]. Conceivably, it would be particularly interesting to assess whether substituting rofecoxib by another COX-2 inhibitor that has not been withdrawn in this combinational regimen yields similar benefits.

Unfortunately, clinical data indicate that usage of pioglitazone may jeopardize health since it has been found to increase the incidence of bladder cancer in DM type II patients [28,148]. For those individuals that have received pioglitazone for a period of >24 months the risk is even higher, as evidenced by a retrospective cohort analysis and meta-analysis [149,150]. Yet, another study argues that further research is needed to allow researchers to make safe inferences regarding the use of pioglitazone and risk for bladder cancer [151]. According to a more recent meta-analysis of randomized trials, pioglitazone is not associated with an overall risk for histologically different types of neoplasms. Instead, pioglitazone decreases the incidence of breast cancer [152] while short-term administration of pioglitazone does not affect the risk for bladder cancer [153].

Οngoing clinical experimentation (e.g. NCT00780234) may hopefully decipher the relationship between pioglitazone and the risk of organ-specific carcinogenesis or even overall neoplasia before an inference can be drawn regarding the anti-cancer or cancer-promoting function of this anti-diabetic agent. In fact, numerous clinical trials are currently active both for pioglitazone and metformin. 

Table 1 indicatively summarizes some of these trials with different types of therapeutic interventions and different histological types of malignancy. This highlights the tremendous research interest regarding the repurposing of these drugs in oncology.

### 2.3. Considerations for Using and Repurposing Metformin and Pioglitazone

**Figure 2:**
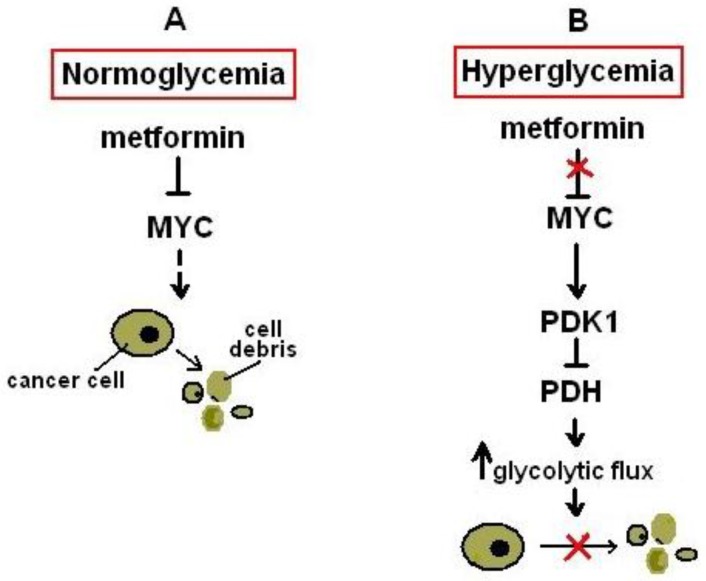
Pathways associated with the sensitivity or resistance of tumor cells to metformin under normoglycemic or hyperglycemic conditions. (**A**) Under normoglycemic conditions metformin opposes the expression of MYC and tumor cells display sensitivity to metformin-induced killing. (**B**) Under hyperglycemic conditions metformin-induced prevention of MYC expression is abolished. MYC upregulates the expression of pyruvate dehydrogenase kinase 1 (PDK1), which in turn inhibits pyruvate dehydrogenase. Glycolytic flux is therefore increased and tumor cells display resistance to the toxic effects of metformin. The red “X” symbol denotes inhibition of a signaling pathway. Upward pointing arrow denotes upregulation.

Metformin, unlike other anti-diabetic drugs such as sulfonylureas, is devoid of major adverse effects such as drug-induced hypoglycemia or lactic acidosis that is more commonly caused by the biguanide phenformin [154]. Would metformin be suitable as an antitumor agent under normoglycemic conditions? According to recent experimental evidence, it is normoglycemia that renders low-dose metformin effective in killing cancer cells. Actually, hyperglycemia seems to favour MYC-dependent enhanced aerobic glycolysis (Figure 2) and tumor cell survival in the presence of metformin (a suppressor of MYC expression), at least in a murine model of ovarian cancer [10]. These data pay credit to the applicability of repositioning metformin in cancer therapeutics particularly in non-diabetics. However, it is well-known that metformin exhibits prosenescent effects and cellular senescence has been linked to various pathologies [155,156,157]. Hence, the impact of metformin’s prosenescent function on overall health merits future investigation by clinical trials assessing the anti-tumor activity of metformin.

Another issue of concern is that at least in mouse models of lung cancer metastasis there is evidence supporting a dichotomized behaviour of PPARγ that inhibits cancer cell invasiveness but on the other hand, it fuels the pro-tumorigenic function of non-malignant cells of the myeloid lineage upon its activation [158]. Further, as it was mentioned above, the usage of pioglitazone has been incriminated for an increased risk of bladder cancer incidence [28,148,149,150]. Although it is seemingly bizarre, a plausible scenario is that pioglitazone may engage an anti-tumor PPARγ/PPRE-dependent pathway only under “permissive” cellular conditions. Such a striking paradigm is provided by the study from Annicotte and colleagues [112] where the presence of the HDAC inhibitor valproic acid is a prerequisite for pioglitazone-mediated anti-metastatic activity in prostate cancer in mice [112]. In other words, pioglitazone in conjunction with valproic acid or another HDAC inhibitor would possibly not exert such pro-tumorigenic effects. This notion is corroborated by the known role of HDAC inhibitors as anti-tumor agents [159,160,161] and warrants further investigation.

### 2.4. Pitfalls and Limitations Stemming from the Use of Mouse Models to Study the Effects of Anti-Diabetic Drugs on Human Cancer Biology

It should be noted that although animal models that have been used successfully to recapitulate many aspects of human carcinogenesis, there are several limitations [162]. Characteristically, the (MMTV)-ErbB2 transgenes recapitulating human breast carcinogenesis carry the viral MMTV-LTR promoter in order to ectopically express the ErbB2 oncoprotein in mammary epithelium (ErbB2 is amplified in up to 30% human breast cancers). MMTV may be accidentally integrated within the int-5/aromatase gene, thereby leading to overexpression of the gene product. However, only a subset of human breast tumors overexpress the human homologue of int-5. Another issue is that the (MMTV)-ErbB2 model develops ER alpha (ERα)-negative tumors; something which is physiologically relevant only for ~50% of human breast tumors [163]. The importance of this is also highlighted by the fact that ER-positivity has been associated with cross-resistance to metformin and tamoxifen upon the prolonged exposure of human breast cancer cells to metformin, as previously mentioned [67]. In addition, human breast tissue is more fibroblastic in comparison with mammary tumors while malignant cells from mammary tumors display a different metastatic pattern compared to human breast cancer cells [163].

Further, there are well-characterized bioenergetic inter-species differences [164] as well as differences at the level of regulation of endocrine function [165]. In the latter case, the role of the leptin system is critical [165] while it is widely known that leptin signalling influences AMPK activity [166]. Besides, there has been reported an interplay among leptin and PPARγ [167,168]. Overall, all these issues should be seriously taken into consideration when mouse models are employed to study the effects of metformin or pioglitazone on cancer biology.

## 3. Conclusions and Future Perspectives

The aforementioned “atypical” antitumorigenic agents would not displace classical chemotherapy regimens but they could possibly augment their efficacy at as low doses as possible, by virtue of new combinational schemes. In addition, many of them exhibit chemopreventive value. The data obtained by clinical studies so far are very encouraging towards this direction. These non-antineoplastic drugs should not been considered as “novel” antitumoral compounds since most of them have been introduced within the market for more than a decade. Consequently, a multi-year clinical experience has been obtained regarding their use; at least dosing and mode of administration for their current therapeutic index. From this point of view, they don’t belong to the same category with hundreds of synthetic or physically isolated compounds which are daily evaluated for their antitumor activity with no former knowledge by clinicians. Further, since these drugs exhibit potent dual therapeutic indices, proper dosing/administration schedule could achieve multiple therapeutic benefits in the exhausted organism of cancer patients (cancer cachexia) which can’t be overloaded with pharmacological agents. Hence, their potent future use in oncology seems quite advantageous. Hopefully, the above-mentioned agents could alter the status quo of cancer therapeutics. This is consistent with the fact that drug repurposing is an emerging concept.

Inarguably, more phase II and phase III clinical studies are needed to assess the anti-cancer properties of metformin and its applicability at the clinical setting far beyond the prevention of breast/colorectal cancer as well as to extirpate any doubts regarding the double-edged sword role of pioglitazone in cancer. The completion of several ongoing clinical studies (e.g. NCT01627067 and NCT00780234) is much-awaited.

The major goal is the design of therapeutic schemes with maximization of the efficacy and minimization of the adverse effects. Researchers and clinicians should always bear in mind that the clinical goal is a reasonable benefit-hazard ratio. Experimentally, pharmaceutical compounds could be considered as molecular “probes” revealing a perplexed cellular microworld which can be molecularly manipulated by them. At the clinical level, they could possibly control pathological processes including tumorigenesis and target their root causes. The experimental results from pharmacological intervention in animals should be extrapolated to humans with great caution. Exploiting the already available knowledge of approved non-antineoplastic drugs against cancer could save precious time from bench to bedside and pave the road for alternative efficacious medical practices.

## Figures and Tables

**Table 1:** Selected Active or Completed Clinical Trials on metformin and pioglitazone for cancer therapeutics or cancer prevention.

Setting	Anti-diabetic Intervention	Other Intervention	Phase	Status	ClinicalTrials. Gov Identifier
Metastatic or unresectable solid tumor or lymphoma	Metformin	Temsirolimus	I	Completed ^§^	NCT00659568
Li Fraumeni Syndrome	Metformin	-	I	Recruiting	NCT01981525
Advanced cancers	Metformin	Temsirolimus	I	Recruiting	NCT01529593
Hormone-resistant prostate cancer	Metformin	Enzalutamide/Laboratory biomarker analysis	I	Not yet recruiting	NCT02339168
Locally advanced or metastatic prostate cancer	Metformin	-	II	Active, not recruiting	NCT01243385
Breast cancer prevention in obese /overweight premenopausal women with metabolic syndrome	Metformin	Placebo	II	Recruiting	NCT02028221
Non-small cell lung cancer (NSCLC)	Metformin	Placebo/Stereotactic body Radiotherapy (SBRT)	II	Recruiting	NCT02285855
Colorectal and breast cancer	Metformin	Exercise training/Exercise training plus metformin	II	Active, not recruiting	NCT01340300
Bladder cancer	Metformin	Simvastatin	II	Not yet recruiting	NCT02360618
Advanced stage ovarian, fallopian tube and primary peritoneal cancer	Metformin	Combination chemotherapy/Laboratory biomarker analysis	II	Recruiting	NCT02122185
Locally advanced NSCLC	Metformin plus chemo-radiotherapy	Chemo-radiotherapy	II	Recruiting	NCT02115464
Metastatic pancreatic cancer	Metformin	Modified FOLFOX 6/ Laboratory biomarker analysis	II	Recruiting	NCT01666730
Hormone-dependent prostate cancer	Metformin	Aspirin/Placebo/ Laboratory biomarker analysis	II	Recruiting	NCT02420652
Metastatic breast cancer	Metformin	Placebo	II	Recruiting	NCT01310231
Castration resistant prostate cancer	Metformin	Enzalutamide	II	Not yet recruiting	NCT02640534
Hormone receptor positive metastatic breast cancer in postmenopausal women	Metformin	Everolimus/Exemestane	II	Active, not recruiting	NCT01627067
Locally advanced rectal cancer	Metformin	-	II	Recruiting	NCT02437656
Breast cancer prevention	Metformin	Placebo	II	Recruiting	NCT02028221
Prostate cancer	Metformin plus bicalutamide	Bicalutamide	II	Recruiting	NCT02614859
Prostate cancer, Prostate cancer recurrent	Metformin	-	II	Recruiting	NCT02176161
Ovarian, Fallopian tube, and Primary peritoneal cancer	Metformin	-	II	Recruiting	NCT01579812
Refractory colorectal cancer	Metformin	Irinotecan	II	Not yet recruiting	NCT01930864
Early stage breast cancer	Metformin	Placebo	III	Active, not recruiting	NCT01101438
Prostate cancer	Metformin	Placebo	III	Recruiting	NCT01864096
Advanced solid tumors	Pioglitazone	Carboplatin	I	Active, not recruiting	NCT02133625
PAX8-PPARγ fusion gene-positive thyroid cancer	Pioglitazone	-	II	Recruiting	NCT01655719
Pancreatic cancer	Pioglitazone	-	II	Recruiting	NCT01838317
Oral leukoplakia	Pioglitazone	Placebo/Laboratory biomarker analysis	II	Completed^§^	NCT00951379
Lung cancer chemoprevention	Pioglitazone	Placebo/fluorescence bronchoscopy/quantitative high resolution computerized tomography (CT) scan	II	Active, not recruiting	NCT00780234
Squamous cell cancer chemoprevention	Pioglitazone	-	II	Enrolling by invitation	NCT02347813

^§^ No results from these studies have been posted yet.
